# Peer Review: How much weightage should the editor give to reviewer’s opinion?

**DOI:** 10.12669/pjms.345.16322

**Published:** 2018

**Authors:** Shaukat Ali Jawaid, Masood Jawaid

**Affiliations:** 1 Shaukat Ali Jawaid Chief Editor, Pakistan Journal of Medical Sciences, Karachi - Pakistan; 2 Masood Jawaid Associate Editor Pakistan Journal of Medical Sciences, Karachi - Pakistan

Finding a good quality reviewer, retaining them and setting up an efficient peer review system are some of the important challenges which the editors are faced universally.^1^ Things are changing so fast that it is becoming extremely difficult to keep pace with new developments in peer review and often one is confronted with a dilemma as to how much weightage an editor should give to the opinion of reviewers?

Although in the historical perspective the editorial peer review developed slowly and haphazardly but it became institutionalized only in 1940.^2^ Internal and external peer review was put into practice by the journals during 18th century to assist the editors in selection of manuscripts for publication. In the past since most of the journal contents used to be written by the editors themselves, they were not interested in peer review. Stephen Lock published his first book on Peer Review titled “A difficult Balance” in 1985. Later the first congress in peer review was held in Chicago in 1989 and since then these peer review congress are being held regularly after every few years in different countries. 2

In the developing low income countries including Pakistan, research culture has not developed much though the situation is now changing for the better. The contribution of these countries to the overall medical literature remains very negligible, hence the process of peer review has also progressed very slowly. Once we asked a senior faculty member in a medical university, “can I send you a manuscript for peer review? His response was, Yes I will pass it on to my resident”. In another case a manuscript was sent for review to the head of a tertiary care institution after he agreed to review it. Later enquiries revealed that he had passed it on to one of his juniors for review and the quality of the review when it was received was also lazy and left much to be desired. This just reflects the attitude of senior faculty members who are too busy and have least interest in such academic activities. There are numerous studies which show that young researchers and those affiliated with teaching institutions are better reviewers.^3^

It was in 1970 that the concept of external peer review blossomed in cardiology. This resulted in critique of papers by outsiders of equal competencies. Dack, the editor of American Journal of Cardiology started sending papers for external review. However, he considered the feedback as advice rather than an authoritative word. Once he is reported to have rejected three papers which had received positive reviews and when he was questioned about his decision, his response was “I read every word of every paper and every review? I know the reviewers, strengths, weaknesses and biases. I ask for their opinion and I am not asking them for their vote. I am the editor, this is my journal; I make the decisions and I take the responsibility”.^4^

There is no denying the fact that peer review does improve the quality of the manuscripts and also provide useful expert opinion to the editors which helps them in making the final decisions whether to accept or reject the manuscripts but it is not without flaws and disadvantages. Some researchers now even question whether the peer review has failed to achieve its objective.

Peer Review could be single blind, double blind and open peer review is the latest and considered the best though all these systems have their own advantages and disadvantages. Richard Smith the former Editor of BMJ felt that open peer review would be more useful. BMJ was initially reluctant to opt for it but later on it was proved that Richard Smith was right. Hence BMJ started practicing open peer review system when Richard Smith was its Editor.^2^

Another recent idea of review adopted by few journal is OE system, in which papers are evaluated post-publication in an ongoing fashion by means of open peer review and rating. Through signed ratings and reviews, scientists steer the attention of their field and build their reputation. Reviewers are motivated to be objective because low-quality evaluations will negatively impact their reputation.^5^

Like editors, the reviewers can also be biased, jealous, ignorant, incompetent hence their reviews can be unreliable, unfair and fail to validate or authenticate. Some researchers also feel that peer review tends to block work which is innovative or contrary to the views of the reviewer thus causing un-necessary delay in publication. Open peer review does make the reviewers more accountable but it has the drawback that junior reviewers may fear reprisal by the established authors, it favour established authors, and it may create resentment and animosity and may result in higher acceptance rate.^2^

However, it is important that the editors should be receptive to new ideas, innovations that has scientific merit and provide them platform to dissiminate. The editors must use the reviewer’s comments as an advice which is not at all binding because it is not uncommon to receive some lazy reviews and editors must tell the reviewers about that. Readers for long have trusted journals to be reliable source of information but the situation has changed a lot during the last four five decades. The trust that physicians used to place in journals has evaporated since the peer review process does not work anymore the way it was expected to be?

It is now widely believed that academic medicine is also getting polluted with the every passing day. In the past the researchers were blamed to receive financial incentives to give positive results of research projects, clinical trials and suppress negative results.^6^ However, more recently the shocking revelations that many editors of the most prestigious medical journals in United States receive payments from the Pharma or medical device industry has questioned the integrity of the editors themselves. It has been reported that only 30% of these journals make it clear to their readers about their policies regarding such conflict of interest. This raises an important question regarding independence and objectivity of these medical journals which are considered as the primary source of information for healthcare professionals in particular and public at large.^7^

Many reviewers do not have enough time to read each paper sent to them carefully, sometime they pass it on to their other colleagues, at times juniors or even residents. It is almost impossible to find good reviewers these days. Sometimes the reviews received are superficial and not helpful. Some reviewers may be spending just a few minutes and then write the report and when the reviews come too late, the editors are under pressure to accept their opinion even if they are inadequate and biased. In such circumstances some editors may be reluctant to overrule the reviewers fearing that they might refuse to review the paper again. Many editors also have a desire to keep the reviewers happy.^2^

At the same time, many new journals or those who have not yet been recognized by their respective country’s regulatory bodies attract very few submission and they are all the times struggling to fill up their pages. Hence they will accept whatever they get irrespective of the quality of paper. Some believe that now peer review remains just a formality. Peer review is dead as it no longer achieves the objective. Posting on a preprint server may replace the traditional peer review process entirely;^4^ hence the responsibility of distinguishing quality has now shifted from the editors to the readers. Reading a paper critically is a great responsibility. Studies have also showed that the priorities of reviewers in basic sciences and those with clinical background differ markedly in some respects. This has an impact on the way they review the manuscripts. Hence, it is considered essential that the reviewers should be provided structured guidelines to maintain uniformity and reduce discrepancy of attitudes between reviewers of diverse background.^8^

Another study by Jawaid SA et al showed that performance of reviewers and quality of their review was mostly dependent on their interests in academics. Best reviewers were retired medical teachers personally known to the editor, those who had attended some training course/workshop on peer review. Editors tend to overuse the efficient reviewers which can lead to burnout syndrome which will then affect the quality of review.^9^

Yet another study reported that the authors, editors and reviewers should have the common goal of enhancing the quality of the communications. It can be achieved if the trio establishes an ethical and professional alliance free of bias and monetary gains and interest- an emerging trend among all those involved in the publication of research.^10^

In view of all this changing scenario the greater responsibility lies with the editors who must critically analyze and review the reviewers’ comments and suggestions. It is the quality of their review which should eventually guide the editors how much weightage they should give to the reviewers’ comments and suggestions while taking a final decision to accept or reject a paper. The decision will vary from reviewer to reviewer.

## References

[1] Jawaid SA (2016). How to find, grow and retain Good Reviewers:An experience from Pakistan. International Journal of Innovation in Science and Mathematics. January.

[2] Jawaid SA, Jafary MH (2002). Peeer Review to improve quality of manuscripts:Are we ready for Open Peer Review?. Pak J Med Sci.

[3] Rennie D, Godliee F, Jefferson T, Editorial Peere Review:its development and ratial (1999). Peer Review in Health Sciences, London. BMJ Books.

[4] Packer M Does Journal Peer Review matter anymore?.

[5] Kriegeskorte N (2012). Open evaluation:a vision for entirely transparent post-publication peer review and rating for science. Front Comput Neurosci.

[6] Ensuring integrity of research remains an uphill task.

[7] Michael Joyce Why we should care that many editors of top medical journals get healthcare industry payments.

[8] Baig S, Ahmed S, Antique H (2016). Reviewing a manuscript:disparity amongst peer reviewers priorities form basic health sciences and clinicians. J Coll Physicians Surg Pak.

[9] (2006). Characteristics of Reviewers and quality of reviews:A retrospective study of reviewers at Pakistan Journal of Medical Sciences. Pak J Med Sci.

[10] Ghani F (2006). The authors, editors and reviewers ethical triad;its impact on quality of papers, contents of journal and image of science. J Pak Dent Asoka.

